# The Proliferative and Apoptotic Landscape of Basal-like Breast Cancer

**DOI:** 10.3390/ijms20030667

**Published:** 2019-02-04

**Authors:** Sarah Alexandrou, Sandra Marie George, Christopher John Ormandy, Elgene Lim, Samantha Richelle Oakes, C. Elizabeth Caldon

**Affiliations:** 1 The Kinghorn Cancer Centre, Garvan Institute of Medical Research, 2010 Sydney, Australia; s.alexandrou@garvan.org.au (S.A.); s.george@garvan.org.au (S.M.G.); c.ormandy@garvan.org.au (C.J.O.); e.lim@garvan.org.au (E.L.); 2 St. Vincent’s Clinical School, Faculty of Medicine, UNSW Sydney, 2052 Sydney, Australia

**Keywords:** basal-like breast cancer, BLBC, triple-negative breast cancer, TNBC, targeted therapies, cell cycle, apoptosis

## Abstract

Basal-like breast cancer (BLBC) is an aggressive molecular subtype that represents up to 15% of breast cancers. It occurs in younger patients, and typically shows rapid development of locoregional and distant metastasis, resulting in a relatively high mortality rate. Its defining features are that it is positive for basal cytokeratins and, epidermal growth factor receptor and/or c-Kit. Problematically, it is typically negative for the estrogen receptor and human epidermal growth factor receptor 2 (HER2), which means that it is unsuitable for either hormone therapy or targeted HER2 therapy. As a result, there are few therapeutic options for BLBC, and a major priority is to define molecular subgroups of BLBC that could be targeted therapeutically. In this review, we focus on the highly proliferative and anti-apoptotic phenotype of BLBC with the goal of defining potential therapeutic avenues, which could take advantage of these aspects of tumor development.

## 1. Basal-Like Breast Cancers Are a Clinical Challenge

Breast carcinomas are a leading cause of cancer mortality and morbidity worldwide with approximately 2.1 million diagnoses estimated in 2018 [1]. Molecular phenotyping based on gene expression profiling has revealed great heterogeneity among breast cancers. Several distinct molecular subtypes, each associated with different clinical outcomes, have been identified by array and RNA-seq studies, and include: Luminal A and B, *ERBB2* overexpression (the gene for the HER2/Neu protein), and normal breast-like and basal-like breast cancers (BLBCs) [2,3]. BLBCs do not generally express *ESR1* (the gene encoding the estrogen receptor (ER)) or *PGR* (the gene encoding progesterone receptor (PR)) and frequently lack *ERBB2* expression, but do express basal cytokeratins (CK), *KRT5* and *KRT6* [4]. Unfortunately, the general lack of hormone and HER2 receptors makes this breast cancer subtype unsuitable and unresponsive to endocrine and HER2-targeted therapies, such as tamoxifen, aromatase inhibitors, and trastuzamab.

BLBC accounts for up to 15% of breast tumors and is commonly diagnosed in pre-menopausal women under the age of 40, women of African descent, and carriers with defects in the familial breast cancer gene, *BRCA1* [5]. The BLBC subtype is characterized by a shorter survival following progression to metastatic disease compared to luminal subsets. Standard care for patients with BLBC includes surgery followed by post-operative (adjuvant) radiotherapy and chemotherapies (e.g., anthracycline and taxane regimens), often with severe side effects that impact quality of life (reviewed elsewhere [6,7]). Unfortunately, these tumors have a high risk of recurrence via the development of chemoresistance, among other mechanisms [8]. BLBCs also have a higher propensity for cerebral and lung metastases compared to the luminal subtypes [4]. This pattern of dissemination complicates and limits further surgical intervention as well as bringing issues with the diffusion of drugs through the blood brain barrier.

## 2. BLBC: A Heterogeneous Group of Breast Cancers

BLBC is as distinct to other breast cancer subtypes as it is to cancers that originate in different organs [9]. One of the most closely related cancer subtypes to BLBC is high grade serous ovarian cancer (HGSOC) [9], and the significant co-occurrence of both tumor types in patients suggests that they could have a common etiology [10]. Among other similarities, both BLBCs and HGSOCs have high rates of mutation in *BRCA1* and *TP53*, and elevated levels of c-MYC (the transcription factor protein myelocytomatosis oncogene cellular homolog) and AKT (the protein kinase AKT8 virus oncogene cellular homolog) [9]. Mutations in *BRCA1*, the product of which recruits DNA repair complexes to sites of DNA damage, is associated with an increased likelihood of developing breast and ovarian cancer [11]. Strikingly, more than 70% of *BRCA1* mutation carriers are likely to develop early-onset BLBC based on gene expression profiling studies [12]. Dysfunction in the *BRCA1* gene results in ineffective homologous recombination, and in addition, defects in the homologous recombination repair mechanisms can also be present in BLBCs that do not present with *BRCA1* mutation, a concept termed ‘BRCAness’ [13]. Nearly all BLBCs that harbor *BRCA1* mutation also have *TP53* mutation [14]. In mouse models, concurrent *Tp53* and *Brca1* mutations lead to increased tumorigenesis, and these two aberrations may help to precipitate BLBC [15].

**Figure 1:**
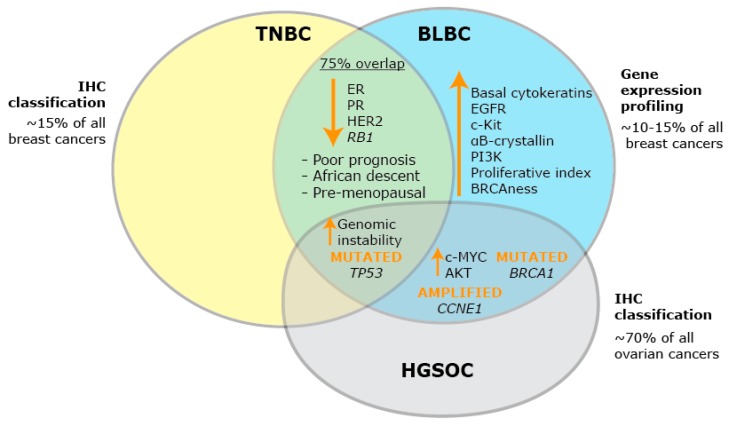
Defining BLBC. Schematic diagram of the defining features of triple-negative breast cancer (TNBC), basal-like breast cancer (BLBC) and high grade serous ovarian cancer (HGSOC). Orange upward arrows indicate an increase in expression; orange downward arrows indicate a decrease in expression.

While gene expression profiling has helped define the BLBC subtype of breast cancers, this description is not used routinely in the clinic [2]. Clinicopathological classification of breast cancers using immunohistochemistry distinguishes the ER+ and HER2+ subtypes and places those tumors that cannot be defined further into a group that has become known as triple-negative breast cancer (TNBC), based on a low level of immunohistochemical signal for ER, PR, and HER2. Of breast cancers, 10–15% have a triple-negative phenotype, and represent 50% of all breast cancer deaths [16]. TNBC is not a specific subtype based on a positive distinctive marker, and as a result, confusion arises when it is assumed to be so. The immunohistochemical definition of TNBC is often used interchangeably with the gene expression based definition of BLBC, but comparative studies show not all TNBCs have basal-like patterns of gene expression, with a 75% overlap in these definitions [17] (Figure 1). For the purposes of this review, when defining in vitro models of BLBC and TNBC, we have used the molecular classification described by Prat et al. [18].

A more accurate pathological definition of BLBC, with specific reference to groups of cancers within this subtype with distinct clinical behaviors, could permit the development of targeted therapies for this subtype [5]. Several studies have investigated different immunohistochemistry markers to define BLBC [19]. Independent tissue microarray studies have confirmed that breast cancers with high levels of basal CK5/6, found in the myoepithelial layer of breast ductal epithelium, are associated with BLBC [20,21], and CK14 is also present in up to 41% of basal-like tumors [22]. The levels of epidermal growth factor receptor (EGFR) are highly correlated to BLBC, and high expression varies from 39–54% in several studies [19,23]. c-Kit (CD117) is a transmembrane protein that regulates cell survival, proliferation, and differentiation, and c-Kit levels in BLBC are associated with a significantly worse prognosis [24]. Finally, the anti-apoptotic protein, αB-crystallin, is detected in 45–80% of BLBC, and is only rarely detected in other subtypes [25]. Of these markers, combining CK5/6, EGFR, ER, and HER2 has been shown to identify BLBC with 100% specificity and 76% sensitivity [19,26], demonstrating a superior prognostic value than merely classifying BLBC under the TNBC umbrella. The addition of PR, CK14, c-Kit, and αB-crystallin as markers for the basal-like phenotype could lead to advances in the clinical identification of BLBC [5,19].

## 3. Identifying Key Hallmarks of BLBC that May Yield New Targets for Therapy

The poor survival profile and inability of BLBC to respond to anti-estrogen or anti-HER2 therapies has led researchers to search for BLBC driver mechanisms that could be targeted with new therapeutic regimes. Hampering this search is the fact that the genomic characterization of BLBC has revealed at least five subsets with basal-like characteristics [27] and significant heterogeneity with multiple potential driver genes and therapeutic vulnerabilities [28]. Through use of the current immunohistochemistry-based classification system for BLBC it will be difficult to implement many of these findings. Molecular markers that are validated in other cancer types will be the exception to this, for example, programmed death-ligand 1 (PD-L1) expression to indicate the application of immunotherapy. Approximately 19% of BLBCs express PD-L1 on associated immune cells [29], and in the recent IMpassion130 trial of advanced TNBC, patients treated with atezolizumab (a PD-L1 antibody) in combination with nab-paclitaxel had a progression free survival of 7.5 months compared to 5 months with nab-paclitaxel alone [30]. While this small subset of BLBC patients may benefit from immunotherapy in the near future, the majority of patients will still receive standard of care chemotherapy.

The complexity of the genomic landscape of BLBC suggests that an important step forward could be to target common phenotypes within BLBC rather than specific molecular aberrations. BLBC has the highest proliferative index of all the breast cancers [3,31], presenting with the highest percentage of Ki67 staining [32,33] and a high mitotic count [34]. This heightened proliferative phenotype is associated with the rapid presentation and growth of BLBC [35]. Excess proliferation can be counteracted by increased apoptosis. Apoptotic cells and heightened caspase-3 activity are commonly detected in BLBC [36,37], but BLBCs have acquired the ability to subvert cell death by engaging numerous anti-apoptotic protective mechanisms. Two of the markers that can be used to identify and define BLBC, *α*B-crystallin and EGFR, have, ultimately, pro-survival outcomes. *α*B-crystallin suppresses the pro-apoptotic protease caspase-3, resulting in resistance to mitochondrial-dependent cell death [23]. Similarly, EGFR plays a role in resistance to cell death as its downstream target, the enzyme, phosphatidylinositol-3-kinase (PI3K), is frequently activated in BLBC [38]. The PI3K/AKT/mammalian target of rapamycin (mTOR) pathway directly promotes survival via modulation of the key members of the B-cell leukemia 2 (BCL-2) family of pro-apoptotic and pro-survival proteins [39]. The deregulated activation of *α*B-crystallin, EGFR, PI3K, and other essential signaling pathways involved in cancer cell survival are a signature hallmark leading to the invasive and apoptotic-resistant phenotype associated with BLBC [23].

In this review, we analyze the growing body of literature that describes the disrupted proliferative and apoptotic pathways in BLBC, highlighting some of the most recent biomarker and molecular studies. We further discuss the potential to apply newly approved therapies that target the cell cycle (e.g., CDK4/6 inhibitors) and apoptosis (e.g., BH3 mimetics). 

## 4. Proliferative Landscape

**Figure 2:**
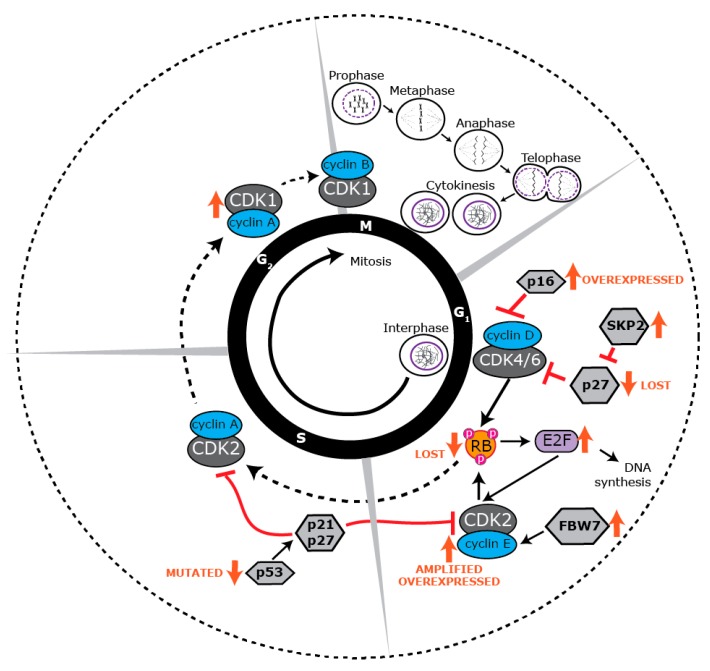
Dysregulation of the cell cycle machinery in BLBC. Schematic view of the cell cycle. Each phase of the cell cycle is regulated by cyclin-dependent kinases (CDKs), their regulatory protein partners (cyclins) and CDK inhibitors. Many proteins in the G_1_/S phase transition of the cell cycle are specifically dysregulated in BLBC. Orange upward arrows indicate an increase in expression; orange downward arrows indicate a decrease in expression.

Cell proliferation is a tightly regulated process that is essential for the growth, development, and regeneration of eukaryotic organisms [40]. Unrestrained cellular proliferation is a fundamental feature of carcinogenesis, which manifests as changes to the regulation of the core machinery that drives proliferation, the cell cycle [41]. The cell cycle consists of two distinct phases: interphase, which comprises G_1_, S, and G_2_ phases, and mitosis (M phase), where cell division occurs. Cell cycle progression is regulated by serine/threonine cyclin-dependent kinases (CDKs) [42] that are activated by cyclins specific to various phases of the cell cycle (Figure 2). Cell cycle entry and proliferation is initiated in the G_1_ phase when CDK4 and CDK6 form heterodimers with D-type cyclins, phosphorylating and inactivating the retinoblastoma (RB) tumor suppressor protein [43,44,45]. The cyclin D-CDK4/6 complex activates E2F transcription factors, promoting the expression of E-type cyclins, which then dimerize with CDK2. Cyclin E-CDK2 complexes further phosphorylate RB as well as other factors essential for DNA synthesis (S phase) [46,47]. During the later stages of DNA replication, CDK2 is activated by cyclin A to facilitate transition into the G_2_ phase. CDK1 subsequently forms a complex with A-type cyclins at the end of interphase to facilitate the onset of mitosis. Cyclin A is degraded following nuclear envelope breakdown in prophase, promoting the formation of cyclin B-CDK1 complexes that are responsible for driving cells through mitosis [48].

## 5. Deregulated Cell Cycle Control in BLBC

BLBC has an increased mitotic index and high rate of proliferation [49], and a major cause of this is the disruption of RB to allow cells to progress without impediment from G_1_ to S phase. RB loss is prevalent across all ER- tumors [50,51] and is particularly high in BLBC, where up to 76% of tumors show loss of heterozygosity (LOH) of the *RB1* gene encoding RB [52]. *RB1* LOH probably acts in concert with the methylation of RB, which is a frequent event across all TNBC [53], and *RB1* LOH in BLBC is associated with worse prognosis [52]. The p16 inhibitor, which selectively inhibits CDK4 and CDK6, is also expressed at high levels in BLBC with RB loss [54]. This is because cells with a compromised RB function initiate a negative feedback loop to inhibit CDK4 with increased expression of the p16 inhibitor. The loss of RB is implicated as an early step in BLBC as lesions of ductal carcinoma in situ often progress to basal-like breast cancer coincident with high p16 and Ki67 [54].

*CCND1* (encoding cyclin D1) is often not amplified and is at lower levels in BLBC [54], consistent with cyclin D1 not being required for phosphorylation of RB [55]; however, *CCND3* (cyclin D3) is frequently amplified [56], and residual disease following chemotherapy of a basal-enriched cohort of TNBC shows amplification of *CCND1*, *CCND2*, and *CCND3*, suggesting that the cyclin D family provides a survival advantage in drug-resistant cancers [57]. Both CDK4 [58] and CDK6 [56,59] are amplified and overexpressed in BLBC, and each is associated with poor overall survival [59]. The high expression of the components of the cyclin D-CDK4/6 complex and their association with poor prognosis is surprising given the canonical role of CDK4 and CDK6 in RB phosphorylation. This may be explained by the discovery of other critical targets for CDK4/6 in breast cancer growth and metastasis. CDK4/6 inhibition in BLBC reduces the CD44+/CD24+ self-renewing population and blocks tumorsphere formation [58], it reduces glucose metabolism [60], and CDK4/6 phosphorylates deubiquitinating enzyme 3 (DUB3) to promote SNAIL-mediated epithelial to mesenchymal transition and metastasis [61].

As well as the loss of RB or increase in CDK4/6 activity, cells find other mechanisms to amplify G_1_/S transition. For example, the E2F5 transcription factor, which is normally released after RB phosphorylation, is significantly up-regulated by amplification in a subset of BLBC, and is associated with an increase in Ki67 staining and shorter disease-free survival [62]. The cyclin E-CDK2 complex contributes to RB phosphorylation as well as promotes the initiation of DNA replication, and multiple mechanisms converge to up-regulate this activity in BLBC. High cyclin E1 expression is characteristic of BLBC, with 26% of BLBCs presenting with elevated levels [63], and other breast cancer subtypes showing relatively low expression [64]. Increased expression may be driven by gene amplification [65] and loss of the cyclin E1 degrader and tumor suppressor protein, F-box and WD repeat domain-containing 7 (FBW7) [66]. High CDK2 activity is also driven by the loss of the CDK2/4/6 inhibitor protein, p27, or gain of S phase kinase associated protein 2 (SKP2), which targets p27 for degradation. Both p27 loss and SKP2 gain are common features of BLBC [63], and linked to poor prognosis [63,67]. Overall, these changes implicate CDK2 activity as an independent driving feature of BLBC, and this is strengthened by the observation that mouse mammary tumors that develop from mouse mammary tumor virus with constitutive Cdk2 expression, have basal-like features [68].

Other enzymes involved in the transition from G_1_/S to G_2_ phase are also elevated in BLBC. The CDC25 dual specificity phosphatases, which function to activate the CDKs, are expressed at high levels in BLBC, and they are predictive of poor prognosis in breast cancer as a whole [69]. Loss of RB correlates with increased CDC25 expression, suggesting that CDC25 up-regulation occurs downstream of RB loss [69]. Topoisomerase IIα, which is critical to DNA replication by cutting coiled DNA, is elevated in BLBC, although it is not predictive of outcome [70,71]. Finally, the c-MYC oncogene, which integrates signaling responses to up-regulate cell cycle progression, is expressed at high levels in BLBC [57], and a signature of genes regulated by c-MYC are also strongly associated with the BLBC phenotype [72].

The high proliferative and mitotic indices of BLBC increase replication stress and mitotic defects, leading to an increase in DNA damage [73]. BLBCs also have a high frequency of *BRCA1* mutation and promoter methylation [74], which exacerbates DNA damage by disabling homologous recombination. A consequence of this is that DNA damage checkpoints are dysregulated in BLBC to protect the cells from excessive cell death. Ataxia telangiectasia and RAD3-related protein (ATR), ataxia telangiectasia-mutated protein kinase (ATM), checkpoint kinase 1 (CHEK1), checkpoint kinase 2 (CHEK2), and G_2_ checkpoint kinase (WEE1) inhibit cell cycle progression into S phase and mitosis following DNA damage, and BLBC often has high CHEK1 [75,76] and CHEK2 [77], presumably giving rise to increased sensitivity to both single-stranded and double-stranded breaks. Lastly, the potent tumor suppressor, p53, which causes G_1_ arrest in response to DNA damage or aberrant oncogene signaling [78], is mutated in 44–82% of BLBC, resulting in abnormal cell proliferation and decreased cell death.

The G_2_/M axis of BLBC also shows significant deregulation, but there is not a consistent association between up-regulated G_2_/M activity and prognosis. This perhaps reflects the generally elevated proliferative capacity of these cancers downstream of core dysregulation by either RB loss or CDK2 activity gain at the G_1_/S transition. The master CDK of G_2_/M, CDK1, is amplified in BLBC [79], but has no relationship with prognosis (C.E.C., personal communication). Mitotic genes that are increased in BLBC and are associated with good prognosis are *BUB1*, *PDZ* associated kinase, and *NIMA* [80], which are involved in centrosome separation and mitotic checkpoints. Conversely, MASTL, a master kinase regulator of mitosis that ensures timely inactivation of CDK1, is high in BLBC and is associated with poor prognosis [81]. Consistent with these observations, knockdown of MASTL will enhance the action of some chemotherapies [82], but not anti-mitotic chemotherapies. b-MYB, which regulates cyclin B1 among other G_2_/M genes, is also elevated in BLBC and is associated with poor prognosis [83].

Overall, BLBC presents with a heterogeneous array of cell cycle defects, but with common themes. The G_1_/S restriction point is side-stepped in these cancers either through depletion of RB or elevation of E2F/CDK2 activity. This leads to a greatly heightened S phase entry, which manifests as increased S phase activity and G_2_/M progression, which is enabled by an array of changes along those axes.

## 6. Targeting BLBC via the Cell Cycle

Chemotherapies have been generally effective in BLBCs as they primarily target highly proliferative cells. Anthracyclines (e.g., doxorubicin; Table 1) target G_1_/S phase of the cell cycle either by preventing DNA and RNA synthesis through intercalation with the DNA or RNA [84]; inhibiting Topoisomerase II to induce DNA damage [85]; generating free oxygen radicals that damage DNA, proteins, and cell membranes [84]; or provoking histone eviction from chromatin, leading to activation of the DNA damage repair pathways or apoptosis [86]. The G_2_/M axis is targeted by taxanes (docetaxel and paclitaxel; Table 1), which disrupt microtubule de-polymerization by reversibly binding to tubulin, resulting in stable microtubules, defects in spindle assembly, chromosomal segregation, and cell division [87,88]. This interferes with mitosis and delays the spindle assembly checkpoint which activates apoptosis [89]. While these treatments are highly effective in BLBC, the use of chemotherapies are associated with extensive cytotoxicity to non-malignant, proliferating cells, presenting as alopecia, nausea, cardiotoxicity [84,90], and neurotoxicity [91].

CDK4/6 inhibitors (palbociclib, ribociclib, and abemaciclib; Table 1) target the G_1_/S transition to produce a cytostatic, anti-proliferative effect in cancer cells. This has led to the successful transition of CDK4/6 inhibitors into the clinic for ER+ breast cancers where it is used in combination with endocrine therapy [92]. This is generally believed to be reliant on an intact RB axis [50], which has been a deterrent to the development of CDK4/6 inhibitor therapy for TNBC or BLBC. Despite this, about 50% of BLBC tumors do present with intact RB [56]. In addition, the CDK4/6 inhibitor, abemaciclib, has demonstrated anti-tumor activity in in vitro models of RB+ TNBC, including BLBC models [93]. In pre-clinical models, CDK4/6 inhibitor therapy has been found to synergize with PI3K/AKT/mTOR inhibitors in HCC-38 TNBC cells [60], which have a strong basal signature [18]. In addition, more recent findings have shown that the non-canonical targets of CDK4/6 activity are important in tumorigenesis, including cancer cell self-renewal, glucose metabolism, and metastasis [58,60,61]. Two studies have suggested that CDK4/6 inhibitor therapy is best targeted at the “luminal androgen receptor” subgroup of TNBC based on its RB status [56,94], but the inhibition of DUB3-mediated metastasis by CDK4/6 inhibition appears to be specifically associated with BLBC [61], as DUB3 can drive a basal-like phenotype [61].

Several clinical trials are assessing CDK4/6 inhibition across breast cancers, allowing for future assessment of applicability in BLBC. NCT03130439 (ClinicalTrials.gov) is assessing abemaciclib as a standalone agent in metastatic RB+ TNBC. Two trials are assessing paclitaxel in combination with palbociclib [NCT01320592] or ribociclib [NCT02599363] in RB+ metastatic breast cancer irrespective of hormone receptor status, and NCT03756090 is assessing the combination of palbociclib with paclitaxel, cyclophosphomide (an alkylating agent), and epirubicin (an anthracycline). The combination of CDK4/6 inhibitors with anti-mitotic therapies, such as taxanes and platinums, has shown promise in pre-clinical models [95], including BLBC models [93], but CDK4/6 inhibitors may potentially antagonize G_1_/S-based therapies, such as anthracyclines [95]. Likewise, co-targeting of G_1_/S and G_2_/M via the combination of alkylating agents with anthracyclines and taxanes showed no benefit in patients [96]. Collectively, these studies highlight the need to apply a degree of caution when considering combination CDK4/6 inhibitor and cytotoxic regimens that rely heavily on cell proliferation for their cytostatic and cytotoxic effects [97].

Inhibition of other cell cycle CDKs has also shown some pre-clinical promise for BLBC. The pan-CDK inhibitor, dinaciclib, inhibits the cell cycle, promoting activity of CDK9 to reduce cyclin B1 expression and cause G_2_/M arrest [98], and it is synthetically lethal in MYC-amplified BLBC, causing both proliferative arrest and apoptosis [99]. The high expression of cyclin E1 in ~26% of BLBC also raises the possibility of CDK2 inhibition to target these cancers [67]. Currently, specific CDK2 inhibitors are not available, although some pan-CDK inhibitors, such as SNS032 and CYC065, do target CDK2 with a higher affinity and have progressed to Phase I clinical trials [100].

Cell cycle inhibition can also be accomplished through cell cycle checkpoint inhibitors (Table 1), providing another potential avenue for BLBC treatment. BLBC has high rates of p53 deficiency, which makes cells highly sensitive to the G_2_ checkpoint that is mediated by WEE1 [101]. WEE1 inhibition forces S phase arrested cells directly into mitosis without completing DNA synthesis, resulting in highly abnormal mitoses and apoptosis. This effect can be exacerbated in TNBC models through the use of CDK2 or ATR inhibition to further increase S phase arrest and increase replication stress [101,102]. Essential mitotic kinases may also provide future targets. For example, polo-like kinase is highly expressed in BLBC, and can be targeted by volasertib [103], which shows some effect in solid tumors, including breast cancer [104].

## 7. Apoptosis: An Essential Process in Healthy Tissues

**Figure 3:**
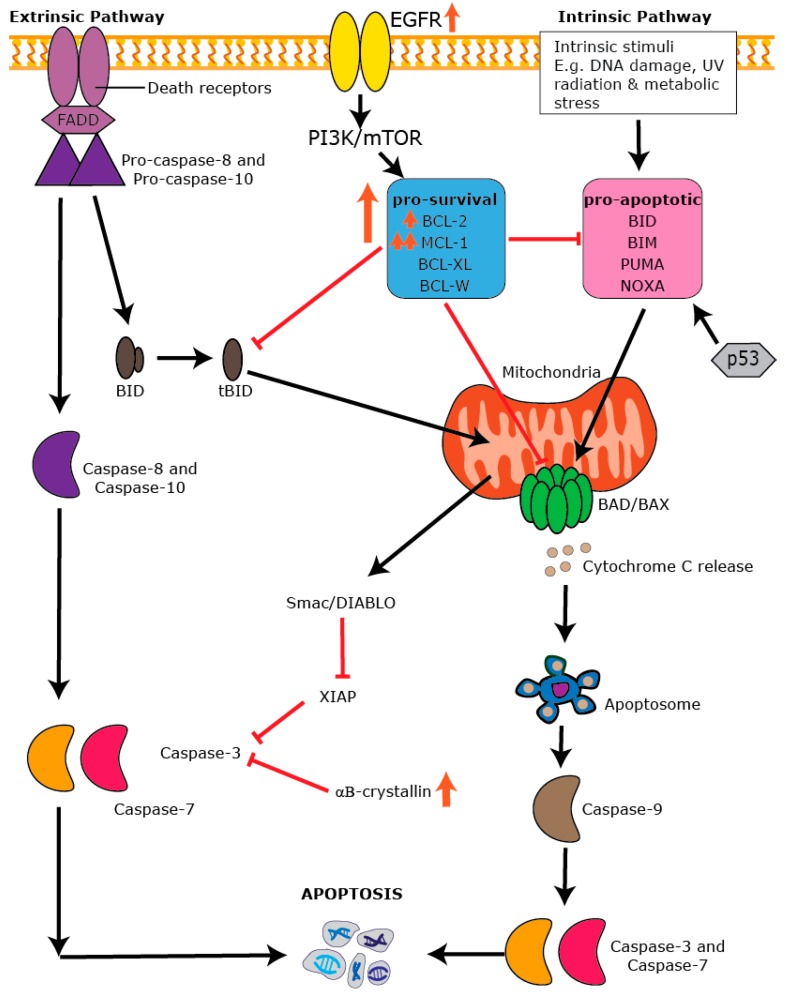
Dysregulation of the apoptotic machinery in BLBC. Schematic view of the extrinsic and intrinsic pathways in apoptosis. Core apoptotic pathways such as EGFR signaling, the pro-survival proteins BCL-2 and MCL-1, and αB-crystallin are dysregulated in BLBC. Orange upward arrows indicate an increase in expression, orange downward arrows indicate a decrease in expression.

Apoptosis is a form of programmed cell death that is necessary for tissue homeostasis, regulation of the immune response, embryogenesis, and the destruction of ageing and dying cells [133,134,135]. It is characterized by a set of distinct morphological characteristics that include membrane blebbing, nuclear and DNA fragmentation, chromatin condensation, and cellular shrinkage [136]. The two main forms of apoptosis leading to caspase activation are the intrinsic (mitochondrial-dependent) pathway and the extrinsic (mitochondrial-independent) pathway [137,138] (Figure 3). The extrinsic pathway is activated by ligand-receptor interactions in the tumor necrosis factor (TNF) superfamily of death receptors containing a death domain that activates caspase-8 at the cell’s surface. The intrinsic pathway is activated by various external stimuli, including growth factor deprivation, stress, ultraviolet radiation, or oncogene activation [139], and is modulated by the BCL-2 family of proteins. The intrinsic pathway is characterized by a cascade of events that lead to increased mitochondria permeability and release of cytochrome C, which binds to apoptotic protease activating factor 1 (APAF-1), leading to the formation of the mature and activated apoptosome that binds and activates the initiator, caspase-9 [136]. Activated caspase-9 then signals the cleavage and activation of caspases-3, 6, and 7, cellular proteases that lead to the destruction of cellular contents. A classic feature of this form of cell death is that it is a non-inflammatory process that produces smaller membrane bound apoptotic bodies that are engulfed by resident phagocytic cells [136].

## 8. The BCL-2 Family and the Intrinsic Apoptotic Pathway

The anti-apoptotic BCL-2 and its related family members (e.g., B-cell leukemia-extra large (BCL-XL), B-cell-like protein 2 (BCL-W), myeloid cell leukemia 1 (MCL-1), and B-cell leukemia 2-related protein A1 (BFL-1/A1)) all contain four BCL-2 homology (BH) domains (BH1-4) with BH domains 1-3 forming a hydrophobic pocket that mediates binding to other BH3-only containing family member proteins (e.g., BID, BAD, BIM, PUMA, NOXA, and tBID). The BH4 domain is highly conserved among the pro-survival members and is essential for providing cell survival signals [140]. The BH3-only proteins are pro-apoptotic, activated in response to cellular stresses (e.g., cytokine deprivation, cytotoxic insult, oncogenic activation), and suppress the actions of the anti-apoptotic proteins resulting in cell death [140,141,142]. BH3-only proteins also directly bind to and activate the pro-apoptotic effectors, BCL-2 homologous antagonist killer (BAK) and BCL-2 associated protein X (BAX), via the binding pocket formed with BH domains 1–3. Once activated, BAX/BAK localize to the mitochondrial surface where they change confirmation, oligomerize, and form pores in the mitochondrial membrane, resulting in loss of permeability and cytochrome release [143].

Crosstalk between the intrinsic and extrinsic apoptotic pathways occurs via the activation of caspase-8 and the cleavage of tBID. The cellular decision to live or die is tightly controlled and one of the most potent inhibitors of cell death is the X-linked inhibitor of apoptosis protein (XIAP), belonging to the family of inhibitor of apoptosis proteins (IAPs) that include Survivin, and cellular (c)-IAP1 and c-IAP2. XIAP directly interacts with caspases-3 and 7 and prevents their activity, resulting in cellular survival (reviewed in [144]). c-IAP1 and 2 inhibit the output of the extrinsic apoptotic pathway via regulation of TNF alpha signaling [145]. Conversely, regulation of IAP activity occurs via DIABLO/second mitochondria-derived activator of caspase (Smac), which are released from the mitochondria subsequent to cytochrome C release in response to cytotoxic stress potentiating cell death [146]. Defects in the ability to execute apoptosis can result from deregulated cell signaling pathways [136], leading to cell survival, a fundamental feature underlying every aspect of carcinogenesis.

## 9. Dysregulation of Apoptosis in BLBC

The pro-survival proteins are induced by multiple growth factor and cytokine signaling pathways that promote cell survival [147], and deregulation of these pathways is a common event in BLBC. The BCL-2 family of proteins can themselves be deregulated, with multiple genetic and proteomic aberrations reported in cancer cells. Some studies have shown that resistance occurs via up-regulation of pro-survival members of the BCL-2 family, including BCL-2 and MCL-1, although BCL-2 is commonly associated with ER positivity and better prognosis in breast cancer, being a favorable prognostic marker [148,149]. The exception is that high BCL-2 levels may be important in drug resistance in BLBC, as it is a significant independent predictor of poor outcome in BLBC patients treated with anthracycline-based adjuvant chemotherapy [150].

The anti-apoptotic protein, MCL-1, is more widely expressed and is present in most BLBCs at varying levels [151,152]. MCL-1 has been shown to have an important role in BLBC cell survival and carcinogenesis [149,153], and also plays a strong role in mediating the survival and chemotherapeutic sensitivity of BLBC and TNBC models [154,155]. MCL-1 protein is normally proteosomally degraded via the ubiquitin ligases, FBW7 and MULE/ARF-BP, leading to its short half-life [156]. Interestingly, dysregulation of FBW7 has been shown to be a prognostic biomarker in breast cancer particularly for ER- and BLBCs with the lowest levels of FBW7 found in these tumor subtypes [157]. Additionally, MCL-1 complexes with MULE are only transiently found in breast cancer cells and this has been shown to play a significant role in increased MCL-1 protein stability [158]. High levels of MCL-1 protein are found in breast cancers independent of the subtype [152], but BLBC has the highest MCL-1 expression, and up to 20% of BLBCs are characterized by genomic amplifications of *MCL-1* [159]. Importantly, a genome wide sensitivity screen reported that BLBCs are largely dependent on proteasome function via proteasomal mediated regulation of the BH3-only protein, NOXA [153], and NOXA preferentially binds to MCL-1 [160]. There is now good evidence suggesting that MCL-1 mediates basal breast cancer cell survival and therapeutic resistance, with several studies showing the importance of this protein in therapeutic resistance where its suppression or antagonism is important for re-sensitization to cytotoxic therapies [146,161,162,163].

High levels of BCL-2 and MCL-1 in drug-resistant BLBC may indicate those cells that are ‘primed for death’ whereby surviving cancer cells with high levels of pro-survival proteins are intrinsically resistant to a wide range of chemotherapeutics, but are, in fact, poised for death when exposed to an agent that competitively antagonizes the elevated pro-survival protein [164]. This likely relies upon the interactions of the pro-survival proteins with the BH3 proteins and apoptotic effectors, which are often coincidentally elevated in cancer cells. This has led some researchers to develop a ‘BH3 profiling’ assay that may be useful to predict dependence on the BCL-2 family of proteins for survival and chemotherapeutic resistance [165]. It remains to be seen whether BH3 profiling could be used to determine dependence on the BCL-2 family for BLBC; nevertheless, the presence of BCL-2/BIM complexes were shown to be important for sensitivity to the BCL-2 antagonist, ABT-737, in patient-derived breast cancer xenografts with basal-like characteristics [152]. Interestingly, an analysis of the METABRIC dataset also showed that high levels of *MCL-1* mRNA expression predicted better overall survival in treated HER2+ and BLBCs. Conversely, in untreated cases, high expression of *MCL-1* mRNA predicted poor outcome [149]. These data suggest that high levels of MCL-1, like BCL-2, could predict those BLBCs addicted to MCL-1 for survival, yet are poised and ready to respond to therapy, either by cytotoxic therapies or targeted treatments.

As discussed above, *α*B-crystallin is a defining feature of a large proportion of BLBCs [166]. Overexpression of *α*B-crystallin promotes epidermal growth factor and anchorage independent growth of immortalized mammary epithelial cells and is associated with poor breast cancer specific survival and resistance to neo-adjuvant chemotherapy [167,168]. Small molecule antagonism of *α*B-crystallin can reduce tumor growth and invasiveness of breast cancer cells in part via its functions to repair misfolded vascular endothelial growth factor signaling [169] (Table 2). However, *α*B-crystallin also directly modulates the output of the intrinsic apoptotic pathway, where serine-59 phosphorylated *α*B-crystallin was shown to directly interact with BCL-2 and prevent its translocation to the mitochondria [170]. *α*B-crystallin overexpression also suppresses the release of cytochrome C from the mitochondria in part via its effects on BCL-2 [171]. Thus, either targeting the pro-survival effects of *α*B-crystallin directly or subverting its effects on BCL-2 mediated cell survival may be an effective strategy in BLBC.

The tumor suppressor and transcription factor, *TP53*, is mutated in approximately 50% of human cancers, leading to increased levels of inactive p53 in cancer cells [172] that has consequences for sustained cellular survival [173]. Importantly, cancers harboring a *TP53* mutation often have intact apoptotic pathway components, providing a therapeutic opportunity independent of a *TP53* driver mutation, particularly as many cytotoxic drugs primarily act via p53 to induce apoptosis, which can be a point of resistance [174]. p53 controls the transcription of BH3-only proteins, such as PUMA, NOXA, and BAX, in response to cytotoxic stress [175]. There has been substantial work in developing small molecule compounds targeting the mutant p53, such as the MDM2 inhibitors (MI-773301 and Nutlins; Table 2), which restrict MDM2 suppression of p53 function [176]. There are, however, still unresolved issues surrounding the development of p53 as a therapeutic target [177], with diseases, such as breast cancer, not benefiting from p53 inhibition, possibly in part due to the vast heterogeneity of the disease as well as the limited efficacy of targeting transcription factor function. In a p53 mutant context, targeting members of the BCL-2 family may provide a better option for those patients with defective or deregulated p53 function by bypassing this network.

## 10. Targeting Survival in BLBC

The essential role of programmed cell death in the carcinogenesis and acquisition of therapeutic resistance has generated great interest in developing drugs that inhibit survival, either via suppressing the output of signaling pathways leading to survival, or indirectly via antagonizing the actions of pro-apoptotic proteins. Indirect inhibition of cell survival can be achieved via suppression of the pathways commonly altered in BLBC. For example, the PI3K/AKT/mTOR pathway is the most commonly deregulated pathway among breast cancers [9], and an extensive effort has been made to manipulate the output of this pathway in BLBC (reviewed recently in [178]). PI3K is also important for *MCL-1* and *BCL2L11* mRNA transcription, whereas mTOR is important for MCL-1 protein translation [179]. Thus, it is no surprise that manipulating the PI3K/AKT signaling axis in BLBC in vitro models has been shown to be effective particularly in combination with the EGFR inhibitor, gefitinib [180] (Table 2).

The potent IAP inhibitory and apoptosis-promoting functions of Smac have led to the discovery and development of Smac mimetics for the therapeutic targeting of cancer (reviewed elsewhere [181]). Although not widely studied in BLBC yet, Smac mimetics can induce death in basal inflammatory breast cancer cell lines and increase the apoptotic potential of the death receptor ligand, TRAIL [182]. Interestingly, Smac and Protein Kinase C delta (PKCδ) interact in basal-like and luminal breast cancer cells, but are dissociated after taxane cytotoxic treatment [183]. Further, activation of PKC synergizes with the Smac mimetic LBW242 in BLBC cell lines [184]. TRAIL agonists have been also shown to induce apoptosis of BLBC cells. For example, the TRAIL agonist, drozitumab, a DR5-specific TRAIL receptor agonist, has been shown to preferentially kill basal and mesenchymal TNBC cell lines [185]. These early pre-clinical studies provide evidence for the potential of targeting the extrinsic apoptotic pathway in BLBC.

As many cancers are also characterized by aberrant activation of intracellular kinase signaling and p53 [186], it is now thought that targeting direct mediators of the cell survival machinery may serve to improve cytostatic effects of oncogenic kinase inhibitors [187]. Several different classes of antagonists have been developed and studied and include peptides, peptide mimetics, and small molecule antagonists (natural and synthetic) with varying affinity and binding profiles for individual BCL-2 pro-survival proteins [188]. Much of the research has focused on small molecules that mimic the actions of the BH3-only pro-survival proteins (BH3 mimetics), which bind to the hydrophobic binding cleft of pro-survival proteins [189] with highly specific binding preferences for individual family members [160], minimizing systemic toxicities in tissues that depend on the BCL-2 family for survival. Greater improvements in crystallography and drug design have now led to a suite of small molecular inhibitors and BH3 mimetics with greater specificity for their targets. These include WEHI-539 targeting BCL-XL [190] and ABT-199/venetoclax targeting BCL-2 (Table 2).

The development of specific antagonists of the BCL-2 family has been challenging, mainly due to the high homology of the BH domains and the incomplete understanding of how these proteins interact in specific cancer contexts [186], but antagonists of the BCL-2 pro-survival pathway have been developed. Venetoclax/ABT-199 (Venclexta) is the first BCL-2 antagonist approved for use in the United States, European Union, and Australia for chronic lymphoid leukemia and small lymphocytic lymphoma. High BCL-2 is normally associated with ER+ breast cancer [151] and the investigation of the efficacy of ABT-199 in clinical trials focused on ER+ disease and showed a preliminary clinical benefit rate of 69% [191]. However, ABT-199 has now been shown to sensitize TNBC xenografts in vivo to doxorubicin [192]. It remains to be determined whether BCL-2 inhibition is effective in the approximately 10% of patients with BCL-2+ BLBC, building on the promise shown in pre-clinical models of BLBC.

In contrast to BCL-2, most triple-negative and basal-like tumors express MCL-1 at varying levels [193], and the presence of MCL-1 is a barrier to sensitivity to BCL-2/BCL-XL inhibition [193]. Recently, there has been extensive research into the development of BH3 mimetics that antagonize MCL-1 [194]. The indole-2-carboxylic acid, A-1210477, developed by AbbVie was first reported in 2015 and demonstrated a high affinity for MCL-1 as a competitive antagonist of MCL-1/BIM. This compound induced cell death as a single agent and synergized with ABT-263 to produce apoptosis in multiple myeloma and small lung cancer cell lines that were dependent on MCL-1 for cell survival [195]. Like earlier work antagonizing BCL-2 and BCL-XL, MCL-1 antagonism can result in cardiac failure, mitochondrial dysfunction, and other systemic toxicities that may be due to on-target hematopoietic toxicities and off-target effects on BCL-XL [194,196,197]. The Servier compound, S63845, showed even greater specificity and more potent activity than existing BH3 mimetics against MCL-1 and could induce cell death in *MCL-1* amplified cell lines dependent on MCL-1 for survival. Importantly, S63845 is well tolerated in vivo, with efficacy against several BLBC cell lines [198]. Inhibiting MCL-1 in BLBC models can suppress metastatic progression [149] and increase TNBC sensitivity to cytotoxic therapy [148,149,199]. It is yet to be determined whether MCL-1 antagonism is effective in clinical trials of BLBCs, but there are currently two Phase I clinical trials of MCL-1 BH3 mimetics in patients with multiple myeloma, a disease with a MCL-1 dependent etiology [200].

## 11. Combination Targeting of Proliferation and Survival

**Figure 4:**
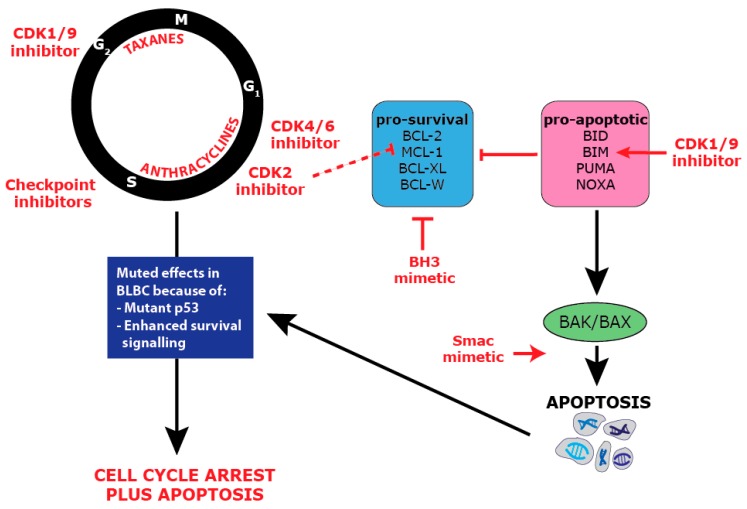
Combination anti-proliferative and pro-apoptotic therapy in BLBC.

Chemotherapies that target critical aspects of cell proliferation, including damaging DNA, rely heavily on cell cycle checkpoints to detect these errors and trigger apoptosis [91,217]. Thus, in BLBC, a subtype of cancer with enhanced proliferation and anti-apoptotic mechanisms, the co-targeting of proliferation and apoptosis could prove particularly successful (Figure 4). Additionally, it has been recognized that cancers with p53 mutation, such as BLBC, often have intact apoptotic cascades, providing another point of weakness [174].

Taxane chemotherapy (docetaxel and paclitaxel) targeted at the G_2_/M cell cycle axis are the standard of care for BLBC, and these have been tested in combination with BH3 mimetics to determine if this will improve efficacy. Docetaxel in combination with the BH3 mimetic, ABT-737, results in a significant improvement in animal survival and tumor growth in BLBC models, but single agent ABT-737 was ineffective at producing apoptosis [152]. Combinations of Smac mimetics and BH3 mimetics were able to increase paclitaxel efficacy in BLBC cell lines, and BH3 mimetics could also re-sensitize paclitaxel-resistant cells to paclitaxel [218]. In TNBC, which broadly overlaps with BLBC, there have also been successes in combining BH3 mimetics with taxanes. The MCL-1 inhibitor, S63845, synergized with docetaxel in a TNBC patient-derived xenograft model to decrease tumor growth [199], and ABT-263 (navitoclax), which targets BCL-2, BCL-XL, and BCL-W, showed synergy with docetaxel in a TNBC cell line [219]. The Smac mimetic, LCL161, also showed promise in a clinical trial testing its combination with paclitaxel in TNBC, where the 30% of patients with an IAP survival signature were responsive to the drug [207]. Consequently, taxane-based therapies appear to combine effectively with a range of anti-apoptotic drugs. This activity could potentially be optimized by tailoring therapy in subsets of BLBC with specific apoptotic defects, for example, *MCL-1* amplified cases.

The other chemotherapies routinely used in BLBC, such as anthracyclines and cyclophosphamide, have for the most part not been tested in combination with pro-apoptotic targeted therapies. A recent study showed that anthracyclines, such as doxorubicin, could synergize with BH3 mimetics in cancers that are “addicted” to BCL-2 family members [220]. Preliminary studies combining cyclophosphamide with a low specificity BH3 mimetic also showed promise in in vitro and in vivo models of B-cell lymphoma [221]. More specific cell cycle inhibitors are yet to be trialed in combination with pro-apoptotic drugs in BLBC. The highly specific CDK4/6 inhibitors have, however, demonstrated pro-apoptotic effects in other cancer types. RB+ non-small cell lung cancer cell lines treated with CDK4/6 inhibitors demonstrated suppression of survival signaling that occurred simultaneously with high Smac and caspase-3 activity [222]. CDK2 inhibition can also be highly effective in inducing apoptosis as it down-regulates the MCL-1 protein, leading to synergy with BH3 mimetics in various cancer cell line models [223].

Pan-CDK inhibitors that target CDK1 and CDK9 show particular promise in BLBC as they can directly induce both proliferative arrest and apoptosis. In MYC amplified TNBC and BLBC models, dinaciclib (CDK1/CDK2/CDK5/CDK9 inhibitor) treatment led to growth arrest, but also enhanced apoptosis through the up-regulation of BH3 protein BIM downstream of MYC [99]. This suggests that a pan-CDK inhibitor may prove effective even in the absence of MYC amplification if it is combined with a BH3 mimetic. This strategy has already proven effective in pre-clinical studies of myeloma cells where the pan-CDK flavopiridol in combination with obatoclax led to decreased proliferation while preventing MCL-1 pro-survival signals and promoting the release of pro-apoptotic BIM [224]. A Phase I trial is now commencing for the CDK2/CDK9 inhibitor, CYC065, in combination with venatoclax, the BCL-2 inhibitor, in relapsed or refractory chronic myeloid leukemia [NCT03739554].

## 12. Conclusions

The current strategies to treat BLBC are non-specific and need to be refined, and a detailed understanding of the molecular mechanisms underpinning this subtype of breast cancer is essential for the introduction of treatment regimens to improve survival. The existing standard of care for BLBC is highly reliant on chemotherapy, and honing in on the underlying mechanisms of these drugs may provide better specificity in the treatment of BLBC. Proliferation is a core pathway targeted by chemotherapy, but without the balanced targeting of cell survival, it is probably not sufficient to merely target cell cycle pathways. This is exemplified by the rapid tumor recurrence that is experienced in BLBC. Of BLBC patients, 38% recur with metastatic disease at an average of 2.3 years, in comparison to ER+ breast cancer, which has a 24% recurrence rate occurring at an average of 4.4 years [225].

Combining anti-proliferative and pro-apoptotic therapies in BLBC is relatively underdeveloped, but the studies to date on combination therapies are highly promising, as many different combinations across these two cancer hallmarks show potential synergy. However, the complex interplay between drugs is a serious consideration in designing new therapies for BLBC. Some targeted therapies benefit from sequential administration in order to access cells in their most vulnerable state, and this is exemplified in a study showing the effectiveness of staggered administration of EGFR inhibitors prior to anthracyclines in BLBC models, as well as other models with high EGFR activity [226].

Finally, an understanding of the heterogeneity within BLBC may also be critical in designing personalized strategies for patients which take advantage of the different proliferative and apoptotic alterations within their cancers. For example, the high rate of *BRCA1* deficiency among BLBC [9] opens the possibility of combination therapy with poly (ADP-ribose) polymerase (PARP) inhibitors. PARP inhibitors already have proven efficacy as monotherapies for *BRCA1*-/- advanced breast cancer, and multiple trials are now assessing possible combinations with cell cycle-based chemotherapies for TNBC (reviewed in [227]). Another recent example is the addition of immunotherapy to nab-paclitaxel in the IMpassion130 trial of TNBC, leading to a significant benefit to those patients with PD-L1 expressing immune cells [30]. Multiple other vulnerabilities, such as proteosomal dependency, NF-κB pathway activation, and BET domain inhibitor sensitivity, have been identified within some BLBC models [28]. As biomarker and clinical tools for these pathways are further developed, these may too prove effective in BLBC, especially when applied in combination with drugs that target the core proliferative and apoptotic pathways.

## Figures and Tables

**Table 1:** Cell cycle directed therapies for BLBC.

Therapy	Drug Examples	Mechanism of Action	Drug Status	Clinical Use	Mode of Administration	Adverse Events
**Chemotherapy**						
**Anthracycline [84,90,105,106,107]**	Doxorubicin–Adriamycin (Bedford Laboratories)	Intercalates with DNA and RNA, prevents DNA/RNA synthesis and induces DNA damage by inhibiting Topoisomerase II Generates free radicals that damage DNA, proteins and cell membranes Induces G_1_/S phase arrest. Effect: cytostatic and cytotoxic	FDA approved	ALL, AML, lymphoma, neuroblastoma, sarcoma, Wilms’ tumor, SCLC, breast, gastric, ovarian, thyroid, bladder cancers	IV	Alopecia, nausea, cardiotoxicity
	Epirubicin–Ellence (Pfizer)			Breast cancer		
**Alkylating Agent:** **Nitrogen mustards [108,109]**	Cyclophosphamide–Cytoxan (Baxter Healthcare Corporation)	Covalent linkage of alkyl groups to DNA (*N*-7 position of guanine), preventing DNA synthesis Arrests in all cell cycle phases, particularly G_1_/S. Effect: cytotoxic	FDA approved	Hematological malignancies, neuroblastoma, retinoblastoma, ovarian, breast cancers	IV	Myelosuppression, nausea, alopecia, hemorrhagic cystitis
**Alkylating Agent:** **Platinum [110,111,112]**	Carboplatin–Paraplatin (Bristol-Myers Squibb Company)	Platinates DNA Covalent binding of platinum to DNA (*N*-7 position on purine residues), causing DNA damage. Induces an S and G_2_/M arrest. Effect: anti-mitotic and cytotoxic	FDA approved	Head and neck, ovarian, lung cancers	IV	Myelosuppression
	Cisplatin–Platinol (Bristol-Myers Squibb Company)		FDA approved	Lung, SCCHN, ovarian, uterine, breast, bladder, testicular cancers		Nephrotoxicity, nausea, vomiting
**Taxane [6,87]**	Docetaxel–Taxotere (Sanofi-Aventis)	Disrupts microtubule de-polymerization Induces a G_2_/M arrest. Effect: anti-mitotic	FDA approved	Breast, prostate, gastric cancers, NSCLC, SCCHN	IV	Neurotoxicity, myelosuppression
	Paclitaxel–Taxol [88] (Bristol-Myers Squibb Company)			Ovarian, breast cancers, AIDS-related Kaposi sarcoma, NSCLC		
**Cell Cycle Inhibition**						
**CDK4/6 inhibitor**	Palbociclib-Ibrance [113] (Pfizer)	Targets CDK4/6 Induces G_1_ phase arrest. Effect: cytostatic and anti-proliferative	FDA approved (in combination with endocrine therapy, or as monotherapy (abemaciclib))	ER+ HER2− breast cancer	Oral	Nausea, diarrhoea, fatigue, neutropenia [114]
	Ribociclib–Kisqali [115] (Novartis)					
	Abemaciclib-Verzenio [116] (Eli Lilly)					Gastrointestinal toxicity [117]
**Pan-CDK inhibitor**	Dinaciclib [99,118,119] (MK7965, SCH727965; Merck & Co.)	Targets CDK1, CDK2, CDK5, CDK9 Induces G_1_ and G_2_/M phase arrest, inhibits phosphorylation of RNA polymerase II and down-regulates *MCL-1*, and is synthetically lethal in MYC-amplified tumors. Effect: cytostatic and cytotoxic	Orphan drug designation	CLL	IV	Hematological and gastrointestinal toxicity [120]
			Investigational compound in clinical trials: Phase III (*n* = 1) Phase II (*n* = 3) Phase I (*n* = 11)	Various advanced solid tumors and hematological malignancies		Toxicity due to lack of efficacy
	Flavopiridol–Alvocidib [118,121] (Sanofi-Aventis)	Targets CDK1, CDK2, CDK4, CDK6, CDK7, CDK9 Induces G_1_ and G_2_ phase arrest, inhibits phosphorylation of RNA polymerase II and down-regulates *MCL-1*. Effect: cytostatic, pro-apoptotic and inhibits transcription	Orphan drug designation	AML	IV	Tumor lysis syndrome, oral mucositis, gastrointestinal toxicity [122]
			Investigational compound in clinical trials: Phase II (*n* = 24) Phase I (*n* = 33)	Various advanced solid tumors and hematological malignancies		Considerable toxicity due to limited efficacy
	SNS032 [123] (Sunesis)	Targets CDK2, CDK7, CDK9 Inhibits phosphorylation of RNA polymerase II and down-regulates *MCL-1* and XIAP. Effect: S phase arrest, pro-apoptotic and inhibits transcription	Investigational compound in clinical trials: Phase I (*n* = 2)	B-lymphoid malignancies [NCT00446342][124]	IV	Myelosuppression
				Various solid tumors [NCT00292864][125]		Fatigue, abdominal pain
	CYC065 [100,126] (Cyclacel Pharmaceuticals, Inc.)	Targets CDK2, CDK9 Inhibits phosphorylation of RNA polymerase II. Effect: S phase arrest, pro-apoptotic and inhibits transcription	Investigational compound in clinical trials: Phase I (*n* = 2)	CLL in combination with venetoclax [NCT03739554] Advanced solid tumors/lymphoma [NCT02552953]	IV	Not yet reported
**Cell Cycle Checkpoint Inhibition**						
**WEE1 inhibitor [101,127]**	Adavosertib (AZD1775, MK-1775; AstraZeneca)	Targets WEE1 Inhibiting WEE1 promotes G_2_ checkpoint escape, premature mitotic entry and DNA damage during S phase. Damaged DNA is not repaired prior to mitosis, encouraging mitotic catastrophe. Effect: pro-apoptotic	Orphan drug designation	Ovarian cancer	Oral	Not yet reported
			Investigational compound in clinical trials: Phase II (*n* = 5) Phase I (*n* = 5)	Various advanced solid tumors and hematological malignancies		
**ATR inhibitor [128,129]**	AZD6738 [101](AstraZeneca)	Targets ATR activity ATR is a DNA damage response kinase, activated by DNA damage. ATR inhibition prevents activation of CHEK1 and the detection of DNA damage and replication stress at the G_2_/M checkpoint.Effect: pro-apoptotic	Investigational compound in clinical trials: Phase II (*n* = 10) Phase I (*n* = 8)	Various advanced solid tumors and hematological malignancies	Oral	Not yet reported
	M6620/VX-970 (Vertex Pharmaceuticals Inc.)		Investigational compound in clinical trials: Phase II (*n* = 6) Phase I (*n* = 7)	Various advanced solid tumors		
**PLK1 inhibitor**	Volasertib [130,131,132] (BI 6727; Boehringer Ingekheim)	Cell cycle kinase inhibitor targeting PLK1, PLK2 and PLK3 Induces pro-metaphase arrest by interfering with mitosis (e.g., entry, spindle formation, chromosome separation and cytokinesis). Effect: anti-mitotic, pro-apoptotic and anti-proliferative	Orphan drug designation	AML	IV	Not yet reported
			Investigational compound in clinical trials: Phase III (*n* = 1) Phase II (*n* = 4) Phase I (*n* = 11)	Various advanced solid tumors and hematological malignancies		Hematological toxicity

Abbreviations: AML: acute myeloid leukemia; ALL: acute lymphoblastic leukemia; ATR: ataxia telangiectasia and RAD3-related protein; CDK: cyclin-dependent kinase; CLL: chronic lymphoid leukemia; DNA: deoxyribonucleic acid; ER+: estrogen receptor positive; FDA: food and drug administration; HER2−: human epidermal growth factor receptor 2 negative; IV: intravenous (injection); MCL-1: myeloid cell leukemia 1; NSCLC: non-small cell lung cancer; PLK: polo-like kinase; RNA: ribonucleic acid; SCCHN: squamous cell carcinoma of the head and neck; SCLC: small cell lung cancer; XIAP: X-linked inhibitor of apoptosis protein.

**Table 2:** Targeting apoptosis and survival in BLBC.

Therapy	Drug Examples	Mechanism of Action	Drug Status	Clinical Use	Mode of Administration	Adverse Events
**Heat Shock Protein Inhibition**						
**αB-crystallin antagonist [169]**	NCI-41356	Targets the interaction between αB-crystallin and VEGF_165_ αB-crystallin repairs misfolded proteins (e.g., VEGF), and αB-crystallin antagonism can inhibit VEGF production in tumor cells. Effect: reduces tumor growth and invasiveness in vitro and in vivo, decreases angiogenesis	Investigational compound in pre-clinical studies	Triple negative breast cancer models	-	Not yet reported
**p53 Inhibition**						
**MDM2 inhibitor**	MI series [201] e.g., MI-77301 (SAR405838; Sanofi-Aventis)	Targets MDM2 Inhibits the MDM2-p53 interaction and activates p53 transcriptional activity. Effect: anti-proliferative and induction of p53-mediated apoptosis	Investigational compound in clinical trials: Phase I (*n* = 2)	Solid tumors	Oral	Not yet reported
	Nutlins [202] e.g., Idasanutlin (RG7388; Roche)		Investigational compound in clinical trials: Phase III (*n* = 1) Phase II (*n* = 1) Phase I (*n* = 9)	Various advanced solid tumors and hematological malignancies		
**Signal Transductor Inhibition**						
**Tyrosine kinase inhibitor [203,204]**	Gefitinib–Iressa (AstraZeneca)	Targets EGFR at the intracellular domain Inhibits the kinase activity of EGFR to promote proliferation and survival. Effect: anti-proliferative and pro-apoptotic	FDA approved	NSCLC	Oral	Gastrointestinal toxicity
**Second mitochondria-derived activator of caspase (Smac) mimetic**						
**Smac mimetics/IAP antagonist [205]**	LBW242 [184,206] (Novartis Oncology)	Targets IAPs Binds to and supresses IAPs (e.g., XIAP, c-IAP1 and c-IAP2), inducing cell death via caspase activation. Effect: pro-apoptotic	Investigational compound in pre-clinical studies	Breast, ovarian cancers, MM, AML, melanoma cell lines	Oral	Not yet reported
	LCL161 [207] (Novartis Oncology)		Investigational compound in clinical trials: Phase II (*n* = 3) Phase I (*n* = 6)	Various solid tumors and hematological malignancies		Gastrointestinal toxicity
**Pro-apoptotic Receptor Agonists**						
**Apo2/TRAIL agonist [208,209,210]**	Drozitumab (PRO95780; Genentech Inc.)	Targets DR5 Binding of TRAIL to DR5 initiates an apoptotic signaling cascade (extrinsic pathway) via assembly of the death induced signaling complex involving caspases-8 and 10. Effect: pro-apoptotic	Investigational compound in clinical trials: Phase II (*n* = 2) Phase I (*n* = 2)	NSCLC, non-Hodgkin’s lymphoma, colorectal cancer	IV	Hematological toxicity, diarrhoea, nausea
**BH3-only Pro-survival Proteins (BH3 mimetics)**						
**BCL-2 inhibitor [190]**	ABT-737 [211] (Abbott Laboratories)	Targets BCL-2, BCL-XL and BCL-W, blocking the interaction with pro-apoptotic BCL-2 family members Effect: BAX/BAK-dependent apoptosis	Investigational compound in pre-clinical studies	SCLC, follicular lymphoma, CLL cell lines	-	Not yet reported
	Navitoclax [212,213] (ABT-263; Abbott Laboratories)		Investigational compound in clinical trials: Phase II (*n* = 4) Phase 1 (*n* = 21)	Various advanced solid tumors and hematological malignancies	Oral	Thrombocytopenia, neutropenia
	Venetoclax - Venclexta [214,215] (ABT-199; AbbVie Inc.)	Targets BCL-2, blocking the interaction with pro-apoptotic BCL-2 family members Effect: BAX/BAK-dependent apoptosis	FDA approved	CLL and SLL		Neutropenia, tumor lysis syndrome
**BCL-XL inhibitor**	WEHI-539 [216]	Targets BCL-XL, blocking the interaction with pro-apoptotic BCL-2 family members Effect: BAK-dependent apoptosis	Investigational compound in pre-clinical studies	Murine associated fibroblast cells	-	Not yet reported
**MCL-1 inhibitor**	S63845 [198] (Servier)	Targets MCL-1 and disrupts the binding of BAX/BAK to MCL-1 Effect: BAX/BAK-dependent apoptosis	Investigational compound in pre-clinical studies	MM, AML, lymphoma cell lines	-	Not yet reported
	A-1210477 [195] (AbbVie Inc.)	Targets MCL-1 and disrupts the MCL-1/BIM protein complex Effect: BAX/BAK-dependent apoptosis		NSCLC, MM cell lines		

Abbreviations: AML: acute myeloid leukemia; BAK: BCL-2 homologous antagonist killer; BAX: BCL-2 associated protein X; BCL-2: B-cell lymphoma 2; BCL-XL: B-cell leukemia-extra large; BCL-W: B-cell-like protein 2; c-IAP: cellular inhibitor of apoptosis protein; CLL: chronic lymphoid leukemia; DR5: death receptor 5; EGFR: epidermal growth factor receptor; FDA: food and drug administration; IAP: inhibitor of apoptosis protein; IV: intravenous (injection); MCL-1: myeloid cell leukemia 1; MM: multiple myeloma; NSCLC: non-small cell lung cancer; SCLC: small cell lung cancer; SLL: small lymphocytic lymphoma; TRAIL: tumor necrosis factor related apoptosis inducing ligand; VEGF: vascular epidermal growth factor; XIAP: X-linked inhibitor of apoptosis protein.
